# Perspectives on PARP Inhibitor Combinations for Ovarian Cancer

**DOI:** 10.3389/fonc.2021.754524

**Published:** 2021-12-15

**Authors:** Renata Colombo Bonadio, Maria del Pilar Estevez-Diz

**Affiliations:** ^1^ Instituto do Cancer do Estado de Sao Paulo, Faculdade de Medicina da Universidade de Sao Paulo, Sao Paulo, Brazil; ^2^ Medical Oncology, Oncologia D’Or, Sao Paulo, Brazil

**Keywords:** PARP inhibitor, combinations, homologous recombination, ovarian cancer, DNA repair

## Abstract

Poly (ADP-ribose) polymerase (PARP) inhibitors constitute an important treatment option for ovarian cancer nowadays. The magnitude of benefit from PARP inhibitors is influenced by the homologous recombination status, with greater benefit observed in patients with BRCA mutated or BRCA wild-type homologous recombination deficient (HRD) tumors. Although some PARP inhibitor activity has been shown in homologous recombination proficient (HRP) ovarian tumors, its clinical relevance as a single agent is unsatisfactory in this population. Furthermore, even HRD tumors present primary or secondary resistance to PARP inhibitors. Strategies to overcome treatment resistance, as well as to enhance PARP inhibitors’ efficacy in HRP tumors, are highly warranted. Diverse combinations are being studied with this aim, including combinations with antiangiogenics, immunotherapy, and other targeted therapies. This review discusses the rationale for developing therapy combinations with PARP inhibitors, the current knowledge, and the future perspectives on this issue.

## Introduction

Almost 50% of ovarian carcinomas have homologous recombination deficiency (HRD) (1). The homologous recombination (HR) is a precise mechanism of double-strand breaks repair, which uses the sister chromatids as a template. Non-homologous end-joining (NHEJ) is another machinery of double-strand breaks repair that predominates when HR is reduced and is more error-prone. Considering this, homologous recombination deficiency compromises DNA repair, conferring a high sensitivity to platinum agents and Poly-ADP ribose polymerase (PARP) inhibitors. The PARP enzyme acts in the base excision repair (BER) mechanism that repairs single-strain breaks. When PARP inhibitors are used, unrepaired single-strain breaks lead to double-strain breaks during replication. With HRD, this results in the accumulation of DNA damage, leading to cell death, which is known as synthetic lethality.

Although germline BRCA mutations are the most well-known cause of HRD, they account for only a part of HRD in ovarian cancer. The highest BRCA mutations frequency occurs in high-grade serous ovarian carcinoma, in which they are identified in 14 – 20% of the cases (1). Germline BRCA mutations are also common in some particular scenarios of other malignancies, such as triple-negative breast cancer and metastatic castration resistance prostate cancer. In the latter one, germline BRCA mutations occur in 11-33% of the cases, which is considerably higher than the occurrence in localized disease (2).

Other causes of HRD include somatic BRCA mutations, germline or somatic mutations in other homologous recombination genes, and possibly methylation of the BRCA promoter. Unfortunately, since mutations of selected genes other than BRCA are rare, accessing its implication on HRD and PARP sensitivity is challenging. On the other hand, HR status can be accessed through the evaluation of the genomic instability that occurs as a consequence of HRD. The HRD-related genomic scar involves three genomic alterations: the loss of heterozygosity (LOH), the telomeric allelic imbalance, and large-scale state transitions. Thus, HRD can be identified by tests that show a LOH ≥ 16% (Foundation Medicine LOH) (3) or an HRD score ≥ 42 (4), which is a score provided by the evaluation of the three genomic alterations (MyChoice ^®^ HRD test – Myriad Genetics).

Currently, PARP inhibitors hold an important role in ovarian cancer treatment, especially as maintenance therapy for platinum-sensitive disease after chemotherapy in the first-line setting or after recurrence (3–8). Four drugs (olaparib, rucaparib, niraparib, and veliparib) had their efficacy as maintenance therapy shown in phase III trials. Talazoparib is another PARP inhibitor with an activity demonstrated in several malignancies, including ovarian cancer. The pharmacokinetics and pharmacodynamics differ between the PARP inhibitors, which may influence their efficacy and tolerability. Talazoparib has an enhanced PARP trapping capability, contributing for its highest potency (9). On the other hand, veliparib has a lower PARP trapping capability, but can be combined with cytotoxic chemotherapy with acceptable tolerability (8).

For three of these drugs (rucaparib, niraparib, and veliparib), patients were included in the phase III trials regardless of BRCA or HRD status. Results showed that platinum-sensitive patients might benefit from PARP inhibitors even if they are BRCA-wild type and homologous recombination proficient (3, 4, 7). Nevertheless, the magnitude of benefit is largely influenced by BRCA and HR status. Undeniably, the benefit with higher clinical relevance is observed in BRCA-mutated or HRD cohorts. For instance, in the PRIMA trial of niraparib maintenance in newly diagnosed ovarian cancer, the improvement in median progression-free survival was 11.2 months in patients with BRCA mutations (HR 0.40, 95% CI 0.27 - 0.62), 11.4 months in those with HRD without BRCA mutation (HR 0.50, 95% CI 0.31 - 0.83), and 2.7 months in those HR proficient (HR 0.68, 95% CI 0.49 - 0.94) (7).

Considering this, enhancing PARP inhibitor activity in HR proficient tumors is warranted. As previously exposed, half of the ovarian carcinoma patients are HR proficient, deriving limited benefit from PARP inhibitors. One rationale is to combine PARP inhibitors with drugs that could lead to a contextual synthetic lethality. With this aim, drugs that interfere directly or indirectly in the HR mechanism are a possibility. Another important potential for PARP inhibitors combinations is to overcome primary or acquired resistance to PARP inhibitors among HRD tumors. Resistance may occur due to diverse reasons as will be discussed here. In this review, we will discuss the rationale for PARP inhibitor combinations, and review published and ongoing studies of PARP inhibitor combinations in ovarian cancer.

## PARP Inhibitor Resistance Mechanisms and Rationale for Combinations

### BRCA Reversion Mutation and HR Restoration

The HR function restoration is one of the main mechanisms implied in resistance to PARP inhibitors. Secondary somatic reverse mutations in BRCA1/2 genes that restore HR have been identified as a mechanism of resistance for both platinum agents and PARP inhibitors (10).

BRCA sequencing showed that reverse mutations restored the gene open reading frame (11). In germline BRCA-mutated high-grade serous ovarian cancer, secondary mutations were identified in 3% of the primary tumors, 28% of the recurrent tumors, and 46% of the platinum-resistant tumors (10).

In a recent meta-analysis by Tobalina et al. 327 patients with BRCA1/2 mutated tumors were evaluated, with reversion mutations identified in 26.3%. The secondary mutations may be due to deletions, insertions, or single-nucleotide variants. Deletions accounted for most of the cases (58.1% in BRCA1 and 77.6% in BRCA2), which may occur as a consequence of DNA end-joining repair mechanisms as suggested by the identification of mutational signatures related to end-joining repair (12).

BRCA1 alternative splice isoforms are another explanation for at least a partial HR restoration. The BRCA1-Δ11q splice variant result in an hypomorphic BRCA1 isoform that lacks most of the exon 11. This isoform can activate RAD51 expression and decrease the sensitivity to PARP inhibitors (13, 14). Finally, epigenetic mechanisms also influence HR restoration. The hypermethylation of the *BRAC1* promoter silence the gene expression, while the reversion of the hypermethylation allows proper BRCA1 expression and HR proficiency (15).

### Loss of 53BP1 Expression and NHEJ Impairment

Alterations in NHEJ have been implied themselves as resistance mechanisms. The 53BP1–RIF1–REV7–Shieldin axis antagonizes BRCA1 and is involved in NHEJ repair. Decreased 53BP1 or REV7 expression enhances HR and has been associated with poor response to PARP inhibitors (16, 17). Double-strand breaks are recognized by the heterodimers Ku70 and Ku80 to initiate NHEJ. They recruit the DNA-dependent protein kinase catalytic subunit (DNA-PKcs) to form the DNA-PK complex, activating its catalytic activity. Other enzyme implied in NHEJ is the DNA ligase IV (LIG4) that mediates the ligation of the broken ends and is stimulated by the X-Ray Repair Cross Complementing 4 (XRCC4) protein (18). Loss of these core NHEJ factors (Ku70, Ku80, DNA-PKcs, LIG4, XRCC4) may impair the NHEJ pathway and favor HR, representing other possible causes of PARP inhibitor resistance (19). These findings highlight the possibility of exploiting new therapies that targets DNA end-joining repair mechanisms to enhance the durability of PARP inhibitors treatment (12).

Another DNA repair mechanism possibly involved in PARP inhibitor resistance involves the BRCA1-A complex. Although BRCA1 is recognized for its major role in HR, this protein has multiple functions regulated by the formation of complexes depending on the cellular context. The BRCA1-A complex includes factors such as RAP80 and Abraxas and acts restricting the end resection, which is an essential initial step for HR repair of double-strand breaks. On the other hand, BRCA1-B and -C complexes counterbalance the BRCA1-A complex and promote end resection (20). Ataxia-telangiectasia mutated (ATM) protein acts initiating HR and, in a preclinical model of *ATM*-mutant cells, disruption of BRCA1-A complex through loss of specific components led to increased HR activity (21).

### Stabilization of Stalled Fork and Cell Cycle Regulation

HR is a complex DNA repair mechanism and several alterations influence its function. The re-establishment of replication fork stability may also lead to PARP inhibitor resistance. During the DNA repair, HR effectors slow the replication fork and facilitate repair. Proteins such as Pax2 transactivation domain-interacting protein (PTIP) and enhancer of zeste homolog 2 (EZH2) participate in the degradation of replication forks. RAD51-antagonist on X-chromosome (RADX) is another protein that inhibits RAD51 and modulates stalled fork protection, with RADX silencing resulting in fork protection. Thus, decreases in PTIP, EZH2 and RADX prevent the degradation and stabilizes the replication fork, facilitating the DNA repair, which may also result in PARP inhibitor resistance (22, 23). Otherwise, the Fanconi anemia (FANC) family of proteins has a role in the fork stabilization to maintain genomic integrity. In BRCA1/2-deficient cells, FANCD2 promotes fork protection and restart, and this protein overexpression is another mechanism associated with resistance to PARP inhibitors (24).

Similar to what is observed with fork stability, modifications in cell cycle regulation may also influence HR, since cell cycles arrest is required to allow DNA repair. For instance, WEE1 is a serine/threonine kinase that causes G2-M cell cycle arrest in response to single or double DNA strand brakes. An increased WEE1 expression is associated with PARP inhibitor resistance (25). In the same way, cyclin-dependent kinase 1 (CDK1) participates in cell cycle progression and interacts with BRCA1. CDK1 inhibition alters BRCA1 function and impairs HR (26, 27). A synergy of the inhibition of PARP and CDK4/6 has also been suggested. In cancer cell lines, the CDK4/6 inhibitor palbociclib induced downregulation of HR genes regulated by MYC resulting in a contextual synthetic lethality when combined with olaparib (28).

Thus, strategies that increase DNA damage, compromise DNA repair, or modify the cell cycle regulation are under investigation to ameliorate PARP sensitivity.

### PI3K/AKT and Other Pathogenic Pathways Activation

Another potential resistance mechanism is the activation of oncogenic pathways that cross-talk with HR. c-MET/HGFR and PI3K/AKT are pathways known to participate in carcinogenesis, and their upregulation may contribute to PARP resistance (29, 30). Preclinical models showed that c-Met-mediated PARP phosphorylation is associated with PARP inhibitor resistance, while c-Met inhibition diminishes HR activity (29, 31).

Similarly, inhibition of PI3K has also been implied in the reduction of HR and the development of a “BRCAness” state (32). PI3K/AKT pathway has a delicate interaction with BRCA1/2. *In-vitro* studies suggest that BRCA1 suppresses AKT and ERK, while defects in BRCA1 might increase the activation of the oncogenic PI3K/AKT pathway (33). PI3K inhibition confers HR impairment, represented by downregulation of BRCA1/2, decreased expression of RAD51, and increased expression of the markers of DNA damage, poly-ADPribosylation and γH2AX (32, 34). Phosphatase and tensin homologue (*PTEN*) gene is a tumor suppressor gene with an inhibitory effect in the PI3K/AKT pathway. Although PTEN loss has been correlated with BRCA1 disfunction, the impact of the PTEN loss in HR repair is still controverse (35). In addition to its role in suppressing PI3K/AKT pathway, PTEN has been linked to an activity in G2/M checkpoint, which is important for proper HR repair (36). Therefore, PTEN loss alters the checkpoint normal function, compromising the time for proper double-strand break repair. Nevertheless, several studies show discrepant data regarding the association of PTEN loss with the expression of RAD51, an important marker of HR activity (36–38).

Clinical trials of PARP inhibitors plus PI3K/AKT inhibitors for ovarian cancer are currently underway. On the other hand, although PARP inhibitor and c-MET inhibitors have a synergic inhibitory effect on ovarian cancer cells (39), these combinations are still on early study phases, and characterization of the safety profile is needed. Thus, for HR proficient tumors and for those that restore HR function as a resistance mechanism, promising combination strategies involve additional blockade of the HR pathway or of these other oncogenic pathways that cross-talk with HR. In HR proficient tumors, these combinations may achieve the goal of a contextual HRD.

### Multidrug Resistance Protein 1 (MDR1) Overexpression

Other potential PARP inhibitor resistance mechanism include increased drug efflux from tumor cells. As with other systemic drugs, drug efflux through increased activity of p-glycoproteins (MDR1) may limit the intracellular concentration of PARP inhibitors (40).

### PARP1 Mutation or Loss of Expression

Finally, modifications of the drug target may impair its activity. *In vivo* and *in vitro* studies showed that PARP1 mutations alter PARP1 trapping and result in resistance to the highly potent PARP inhibitor talazoparib (13). Preclinical models also showed that loss of PARP1 protein expression causes PARP inhibitor resistance (13, 41). The impact of PARP1 mutation or loss of expression in clinical practice still need to be clarified.

**Figure 1:**
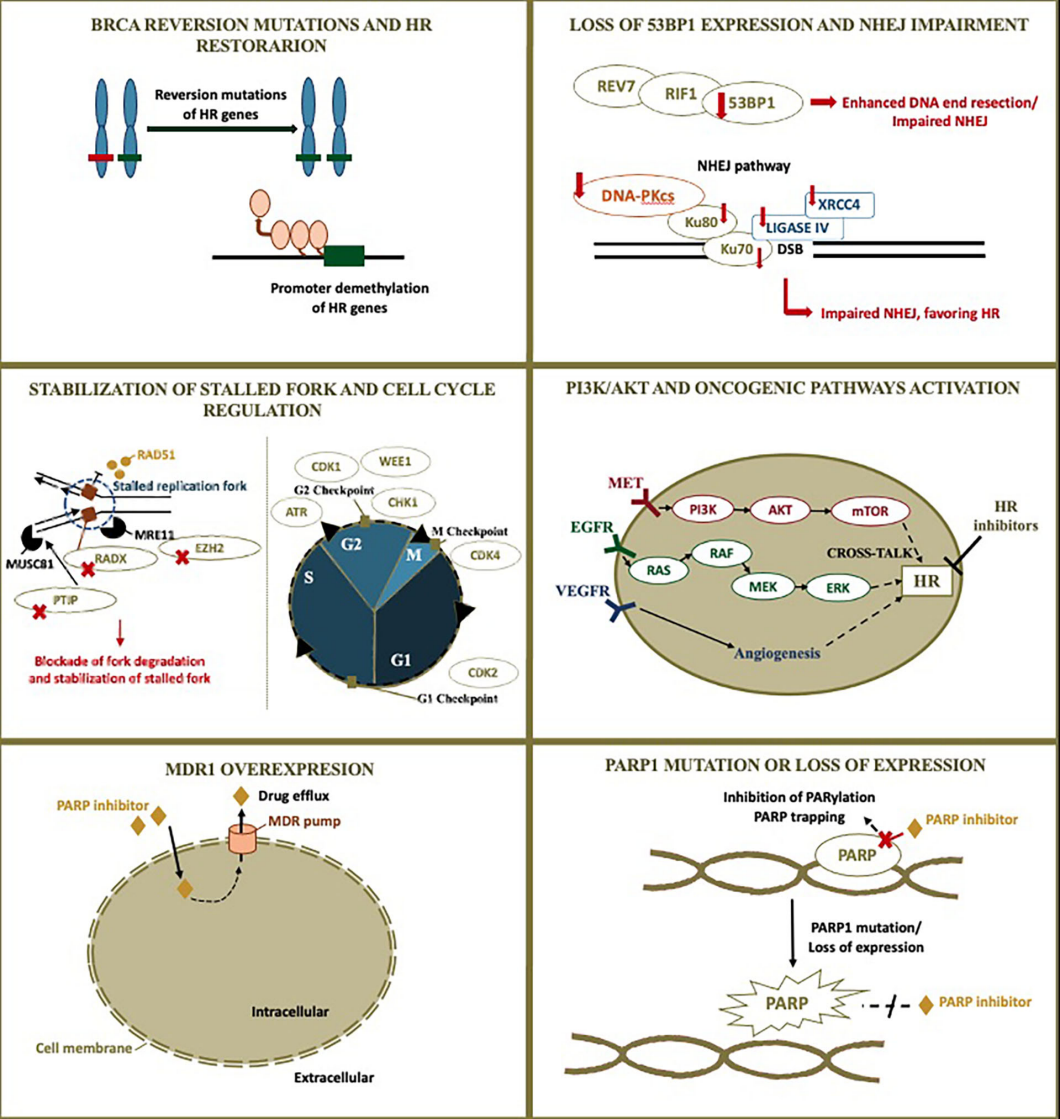
PARP inhibitor resistant mechanism and rationale for combinations. Although homologous recombination restoration due to secondary somatic reverse mutations is well-described as a possible resistance mechanism to PARP inhibitors, many other alterations are also possibly implied. The figure illustrates PARP inhibitor resistant mechanisms and the rationale for combinations currently under investigation in ovarian cancer. Additional blockade of DNA repair may be achieved through targeting other proteins involved in DNA repair, modifying cross-talking pathways to result in a contextual homologous recombination deficiency, and impairing the cell cycle. The following resistant mechanisms are represented: 1) BRCA reversion mutations and homologous recombination restoration; 2) Loss of 53BP1 expression and non-homologous end-joining impairment; 3) Stabilization of stalled fork and cell cycle regulation; 4) PI3K/AKT and other pathogenic pathways activation; 5) MDR1 overexpression; 6) PARP1 mutation or loss of expression. HR, homologous recombination; NHEJ, non-homologous end-joining; PARP, poly (ADP-ribose) polymerase; MDR1, multidrug resistance protein 1.

**Figure 2:**
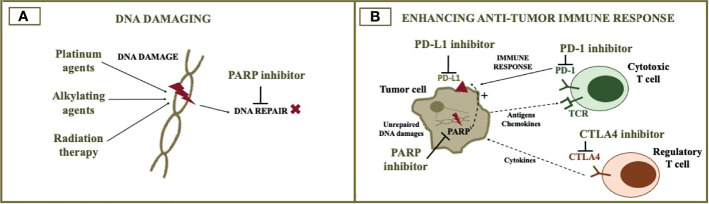
PARP inhibitor combination with cytotoxic agents and immunotherapy. The figure illustrates the rationale for combining PARP inhibitors with DNA damaging agents **(A)** and immunotherapy **(B)**. DNA damaging agents such as cytotoxic chemotherapy and radiotherapy increase DNA damage, when DNA repair is impaired by the PARP inhibitor. The addition of immune checkpoint inhibitors to the PARP inhibitor can potentially optimize anti-tumor immune response. PARP, poly (ADP-ribose) polymerase; PD-L1, programmed death-ligand 1; PD-1, programmed cell death protein 1; CTLA4, cytotoxic T-lymphocyte associated protein 4.

All these possibilities highlight the importance of understanding the underlying mechanism of resistance in the choice of subsequent therapies. For instance, some of the mechanisms may also result in resistance to platinum agents, while others may not. **Figure 1** illustrates PARP inhibitor resistance mechanisms and rationale for therapy combinations exploiting these mechanisms. **Figure 2** illustrates the rationale for combining PARP inhibitors with DNA damaging agents (**Figure 2A**) and immunotherapy (**Figure 2B**). **Tables 1** and **2** summarizes published and ongoing phase II-III studies of PARP combinations for ovarian cancer. The following topic will focus on PARP inhibitor combinations with currently published or ongoing phase II-III trials.

**Table 1:** Published phase II-III studies of PARP inhibitor combinations.

Target	Trial	Year	Phase (N)	Study arms	Population	Efficacy results
**ATR**	Wethington et al. (42) NCT03462342	2021	II (13)	Single arm: O + Ceralasertib	Acquired PARP inhibitor-resistant recurrent OC	ORR 46%
**WEE1**	EFFORT (43) NCT03579316	2021	II (80)	Arm 1: Adavosertib +- O; Arm 2: Adavosertib alone	PARP-resistant OC	Adavosertib + O: ORR 29%, m PFS 6.8 mo; Adavosertib: ORR 23%, mPFS 5.5 mo;
**VEGFR**	Liu et al. (44) NCT01116648	2014	II (46)	Arm 1: Cediranib + O; Arm 2: O alone	Platinum-sensitive recurrent high-grade OC	BRCA wild-type: ORR 76% vs 32% (P=0.006), mPFS 16.5 vs 5.7 mo (HR 0.32, P=0.008); BRCA-mutated: ORR 84% vs 63% (P=0.19), mPFS 19.4 vs 16.5 mo (HR 0.55, pP=0.16)
**VEGFR**	Liu et al. (45) NCT02345265	2018	II (72)	Single arm: O + Cediranib	Platinum-sensitive and platinum-resistant recurrent OC	Platinum-sensitive: ORR 77%, DCR 91%; Platinum-resistant: ORR 20%, DCR 43%
**VEGF**	NSGO-AVANOVA2/ ENGOT-ov24 (46) NCT02354131	2019	II (97)	Arm 1: N + bevacizumab; Arm 2: Niraparib alone	High-grade platinum-sensitive recurrent OC	mPFS: 11.9 vs 5.5 mo (HR 0.35, 95% CI 0.21-0.57); subgroups - HRD: 11.9 vs 6.1 mo (HR 0.38, 95% CI 0.20-0.72); HRP:11.3 vs 4.2 mo (HR 0.40, 95% CI 0.19 - 0.85)
**VEGFR**	EVOLVE (47) NCT02681237	2019	II (34)	Single arm: Olaparib + Cediranib	OC after progression on PARP inhibitor	ORR 12%; 16-week progression-free survival rate 47%
**VEGF**	PAOLA (48) NCT02477644	2019	III (806)	Arm 1: O + Bevacizumab; Arm 2: Placebo + Bevacizumab	Maintenance after first-line therapy for OC	mPFS: 22.1 vs 16.6 mo (HR 0.59, 95% CI 0.49 - 0.72); subgroups - BRCAm 37.2 vs 21.7 mo (HR 0.31, 95% 0.20-0.47); BRCAwt HRD: 28.1 vs 16.6 mo (HR 0.42, 95% CI 0.28-0.66); HRP: 16.6 vs 16.2 mo (HR 1.00, 95% CI -.75-1.35)
**VEGFR**	CONCERTO (49) NCT02889900	2020	IIb (60)	Single arm: O + Cediranib	Platinum-resistant recurrent OC	ORR 15.3%; mPFS 5.1 mo
**VEGFR**	GY004 (50) NCT02446600	2020	III (565)	Arm 1: O + Cediranib; Arm 2: O alone; Arm 3: Platinum-based CT	Platinum-sensitive recurrent OC	mPFS: 10.4 mo with olaparib + cediranib; 10.3mo with platinum-based CT; 8.2 mo with olaparib alone. Olaparib + Cediranib vs CT: HR 0.85, 95% CI 0.66 – 1.11
**VEGFR/PD-L1/CTLA4**	AMBITION (51) NCT03699449	2021	II (70)	Arm 1: O + Cediranib (O+C); Arm 2: Durvalumab + O (O+D)	HRD platinum-resistant recurrent OC	ORR: O+C 50%; O+D 35.7%
**Platinum agent + micro-tubule inhibitor**	Oza et al. (52) NCT01081951	2015	II (168)	Arm 1: O + Carboplatin + Paclitaxel, followed by O maintenance; Arm 2: CT alone	Platinum-sensitive recurrent high-grade serous OC	O plus CT: mPFS 12.2 mo; CT alone: mPFS 9.6 mo (HR 0.51, 95% CI 0.34-0.77, P=0.0012)
**Alkyla-ting agent**	Kummar et al. (53) NCT01306032	2015	II (75)	Arm 1: Cyclophosphamide alone; Arm 2: Cyclophosphamide + V	Previously treated BRCA-mutated ovarian cancer	Cyclophosphamide alone: ORR 19.4%, mPFS 2.3 mo; Cyclophosphamide + Veliparib: ORR 11.8%, mPFS 2.1 mo.
**Topote-can**	Hjortkjær et al. (54) NCT01690598	2018	I/II (27)	Single-arm: Veliparib + Topotecano	Platinum-resistant or partially platinum-sensitive non-BRCA mutated recurrent OC	ORR 0%; CBR 37%; mPFS 2.8 mo
**Platinum agent + micro-tubule inhibitor**	VELIA (8) NCT02470585	2019	III (1140)	Arm 1: V + CT (Carboplatin + Paclitaxel) → V maintenance (V-throughout); Arm 2: V + CT → placebo maintenance (V combination alone); Arm 3: placebo + CT → placebo (control)	First-line treatment for high-grade serous OC	V-throughout: mPFS 34.7 mo; V combination alone: mPFS 21.1 mo; Control: mPFS 22 mo (HR 0.44, 95% CI 0.28-0.68, for V-throughout vs control; HR 1.22, 95% CI 0.82-1.80, for V combination alone vs control)
**PD-L1**	Lee et al. (55) NCT02484404	2018	II (35)	Single-arm: O + Durvalumab	Recurrent (platinum-resistant or platinum sensitive) OC	ORR 11%; DCR 53%
**PD-L1**	MEDIOLA (56) NCT02734004	2019	II (32)	Single-arm: Olaparib + Durvalumab	Recurrent platinum-sensitive germline BRCA-mutated OC	ORR 71.9%; mPFS 11.1 mo
**PD-L1**	MEDIOLA (57) NCT02734004	2020	II (63)	Arm 1: O + Durvalumab (n=32); Arm 2: O + Durvalumab + Bevacizumab (n=31)	Recurrent platinum-sensitive germline BRCA wild-type OC	O + Durvalumab + Bevacizumab: ORR 87.1%; mPFS 14.7 mo; O + Durvalumab: ORR 34.4%, mPFS 5.5 mo
**PD-1**	TOPACIO (58) NCT02657889	2020	II (62)	Single-arm: N + Pembrolizumab	Platinum-resistant or platinum ineligible recurrent OC	ORR 18%; mPFS 3.4 mo
**PD-L1/VEGF**	OPAL (59) NCT03574779	2021	II (41)	Single-arm: O + Dostarlimab + Bevacizumab	Recurrent platinum-resistant OC	ORR 17.9%; mPFS 7.6 mo

PARP, Poly (ADP-ribose) polymerase; OC, ovarian cancer; ORR, overall response rate; DCR, disease control rate; PFS, progression-free survival; mo, months; O, Olaparib; N, Niraparib; V, veliparib; CT, chemotherapy; HRD, homologous recombination deficient; HRP, homologous recombination proficient.

**Table 2:** Ongoing phase II-III studies of PARP inhibitor combinations.

Target	Trial	Phase	Combination	Population
**mTORC1/2 or AKT**	NCT02208375	I/II	O + Vistusertib/O + Capivasertib	Recurrent endometrial, triple negative breast, and ovarian, primary peritoneal, or I/II fallopian tube cancer
**ATR**	NCT03462342 (CAPRI)	II	O + Ceralasertib	Recurrent OC (platinum-sensitive or platinum-resistant).
**ATR**	NCT02264678	I/II	O + Ceralasertib	Platinum-sensitive recurrent BRCA-mutated/RAD51C/D-mutated/HRD OC after progression on a PARP inhibitor
**ATR**	NCT04065269 (ATARI)	II	O + Ceralasertib (AZD6738)	Gynaecological cancers (including relapsed ovarian cancer) with or without ARId1A loss
**ATR**	NCT0257644 (OLAPCO)	II	O + Ceralasertib (AZD6738)	HR-deficient solid tumors
**WEE**	NCT04158336	II	T + ZN-c3	Solid tumors, including OC
**WEE**	NCT0257644 (OLAPCO)	II	O + Adavosertib (AZD6738)	Tumors harboring TP53 or KRAS mutations
**AKT**	NCT0257644 (OLAPCO)	II	O + Capivasertib (AZD5363)	Tumors harboring PTEN, PIK3CA, AKT, or ARID1A mutations or other molecular alterations associated with PI3K/AKT pathway dysregulation
**MEK**	NCT03162627	I/II	O + Selumetinib	Solid tumor, including OC, with Ras pathway alterations, and OC with PARP resistance
**VEGFR**	NCT02340611	II	O + Cediranib	Time OC worsens on O
**VEGF**	NCT03462212 (MITO25)	II	R + Bevacizumab	Maintenance after first-line therapy for high-grade OC
**VEGF**	NCT03326193	II	N + Bevacizumab	Maintenance after first-line therapy for high-grade OC
**VEGF**	NCT02354131 (AVANOVA)	I/II	N + Bevacizumab	Platinum-sensitive epithelial ovarian cancer
**VEGFR**	NCT03278717 (ICON-9)	III	O + Cediranib	Maintenance therapy with O and cediranib or O alone in patients with relapsed platinum-sensitive OC
**VEGFR**	NCT02502266 (COCOS)	II/III	O + Cediranib	Platinum-resistant recurrent ovarian cancer (versus standard chemotherapy)
**VEGFR**	NCT03117933 (OCTOVA)	II	O + Cediranib	Platinum-resistant recurrent ovarian cancer (versus O alone or weekly paclitaxel)
**VEGFR**	NCT02340611	II	O + Cediranib	OC after progression on olaparib alone
**VEFR + PD-L1**	NCT03574779 (OPAL)	II	N + Dostarlimab + Bevacizumab	Recurrent OC
**Alkylat-ing agent**	NCT01113957	II	V + Temozolomide	Recurrent high-grade serous OC
**Topoiso-merase**	NCT03161132 (ROLANDO)	II	O + Pegylated liposomal doxorubicin	Platinum-resistant recurrent OC
**Topoiso-merase**	NCT01012817	I/II	V + Topotecan	Solid tumors, relapsed or refractory OC, or primary peritoneal cancer
**PD-L1/VEG**	NCT03806049	II	N + Bevacizumab ± Dostarlimab	Platinum-sensitive recurrent OC
**PD-1**	NCT03522246 (ATHENA)	III	R + Nivolumab	Maintenance after first-line therapy for OC
**PD-L1**	NCT03602859 (FIRST)	III	N + Dostarlimab	Maintenance after first-line therapy for OC
**PD-L1**	NCT04679064 (MITO33)	III	N + Dostarlimab	Recurrent OC not candidate for platinum retreatment
**PD-L1**	NCT03955471 (MOONSTONE)	II	N + Dostarlimab	Platinum-resistant recurrent OC
**PD-L1**	NCT03651206 (ROCSAN)	II/III	N + Dostarlimab	Maintenance after first-line therapy for ovarian carcinosarcoma
**PD-1**	NCT03740165 (KEYLYNK-001/ENGOT-OV43)	III	O + Pembrolizumab	Maintenance after first-line therapy for BRCA wild-type ovarian carcinosarcoma
**PD-L1**	NCT03737643 (DUO-O)	III	O + Durvalumab	Maintenance after first-line therapy for OC
**PD-L1**	NCT03598270 (ANITA)	III	CT +- Atezolizumab, followed by N +- Atezolizumab	Platinum-sensitive recurrent OC
**PD-L1**	NCT03330405 (JAVELIN PARP Medley)	I/II	T + Avelumab	Locally advanced (primary or recurrent) or metastatic solid tumors, including recurrent platinum sensitive OC
**PD-L1**	NCT03642132 (JAVELIN OVARIAN PARP 100)	III	T + Avelumab	Maintenance after first-line therapy for OC
**PD-L1**	NCT03642132	III	T + Avelumab	First-line therapy for OC
**CTLA4**	NCT04034927	II	O + Tremelimumab	Platinum-sensitive recurrent OC
**CTLA4**	NCT02571725	I/II	O + Tremelimumab	BRCA-deficient OC
**PD-L1/CTLA4**	NCT04169841 (GUIDE2REPAIR)	II	O + Durvalumab + Tremelimumab	Solid tumors, including OC, with mutations of homologous recombination gene
**PD-L1/VEGFR**	NCT04739800	II	O + Cediranib +- Durvalumab	Platinum-resistant recurrent OC
**PD-L1/VEGF**	NCT04015739 (BOLD)	II	O + Bevacizumab + Durvalumab	Platinum-sensitive or platinum-resistant recurrent OC

PARP, Poly (ADP-ribose) polymerase; OC, ovarian cancer; O, Olaparib; N, Niraparib; V, veliparib; R, rucaparib; T, talazoparib; HRD, homologous recombination deficient.

## PARP Inhibitors Combinations

### PARP Inhibitors Plus ATM/ATR/CHK1 Inhibitors

The ATM protein is activated by the presence of double-strand DNA breaks and has a crucial role in initiating HR by signaling to downstream effectors. Ataxia-telangiectasia and Rad3-related (ATR) protein, on the other hand, is activated by single-strand DNA breaks and stressed replication forks. ATR activates downstream effectors, including CHK1 and WEE1, leading to suppression of replication stress and cell cycle arrest. Hence, ATM and ATR are two important DNA repair proteins, which cross talks with each other. Inhibition of ATM, ATR, or CHK1 in combination with PARP inhibitors may therefore improve PARP sensitivity in both HR-proficient and HRD cells (60). Indeed, the combined inhibition of ATR and PARP was able to overcome platinum and PARP inhibitor resistance in patient-derived xenografts models of ovarian cancer (61).

Currently, phase II clinical trials are evaluating these combinations. The CAPRI trial is evaluating the ATR inhibitor, ceralasertib (AZD6738), in combination with olaparib, for platinum-sensitive or platinum-resistant recurrent ovarian cancer (NCT03462342). Preliminary results of CAPRI trial from a cohort of patients with HRD and acquired PARP inhibitor-resistance were recently presented. Results showed an overall response rate of 48% (n=6/13), suggesting that the addition of ATR inhibitor may overcome PARP inhibitor resistance (42). The same combination is also being studied for ovarian cancer patients in OLAPCO (NCT02576444) and ATARI (NCT04065269) trials.

The CHK1 inhibitor, prexasertib, has activity as monotherapy for high-grade BRCA wild-type ovarian cancer, with an overall response rate of 29% (n=8/28) in a phase II trial (62). Interestingly, most patients in the analysis were platinum-resistant. The safety of the combination of prexasertib and olaparib has been shown in a phase I trial (63), but additional studies are still needed to evaluate the efficacy of the combination.

### PARP Inhibitors Plus WEE1 Inhibitors

While WEE1 increased expression may reduce sensitivity to PARP inhibitors, WEE1 inhibition will allow DNA-damaged cells to enter the S-phase, favoring apoptosis and increasing PARP inhibitors efficacy. The WEE1 inhibitor, adavosertib, was studied as a single-agent or combined with olaparib in women with PARP-inhibitor resistant ovarian cancer in the phase II non-comparative EFFORT trial. The study results were recently presented and suggested the efficacy of adavosertib alone and combined with olaparib. Among the 70 patients evaluable for response (35 in each arm), overall response rates were 23% with adavosertib alone and 29% with the combination. Median progression-free survival was 5.5 months and 6.8 months, respectively (43).

One of the cohorts of the multi-arm phase II OLAPCO trial is evaluating the combination of adavosertib and olaparib for tumors harboring *TP53* or *KRAS* mutations (NCT02576444). Patients with p53 loss have the G1/S checkpoint regulation impaired, which may turn them particularly sensitive to WEE1 inhibitors since WEE1 regulates G2/M transition. In a phase II trial, the combination of adavosertib with carboplatin for *TP53*-mutated ovarian cancer after progression to first-line platinum-based therapy had encouraging results, with an overall response rate of 43% (64).

### PARP Inhibitors Plus PI3K/AKT Inhibitors

As previously mentioned, PI3K/AKT inhibition may lead to a “BRCAness” state, downregulating the expression of BRCA and RAD51. Phase I trials have shown the safety of the combination of olaparib with PI3K inhibitors (buparlisib and alperlisib), and preliminary evidence for activity was suggested, with response rates ranging from 29 to 36% in ovarian cancer patients (65, 66). Safety has also been shown in a phase I trial of olaparib plus the AKT inhibitor capivasertib (67). This combination is currently being evaluated in one arm of the phase II OLAPCO trial for tumors with PTEN, PIK3CA, AKT, or ARID1A mutations or other molecular alterations associated with PI3K/AKT pathway dysregulation (NCT02576444). Another phase I/II study is also ongoing of the combination of olaparib with capivasertib or vistusertib (an mTOR inhibitor) for recurrent endometrial and ovarian cancer (NCT02208375).

### PARP Inhibitors Plus Antiangiogenics

Preclinical studies have shown that hypoxia is associated with downregulation of BRCA1 and RAD51, leading to a contextual HRD (68, 69). Decreased expression of BRCA1 and BRCA 2 is also induced by VEGFR3 inhibition (70). These results create the rationale for exploring the combination of PARP inhibitors plus antiangiogenics.

Exciting results were initially presented by a phase II trial that evaluated olaparib plus cediranib, a VEGFR inhibitor, versus olaparib alone for platinum-sensitive recurrent ovarian cancer. The trial showed an improvement in progression-free survival with the addition of cediranib, which was especially relevant in BRCA wild-type tumors (median progression-free survival of 16.5 months with the olaparib plus cediranib vs. 5.7 months with olaparib alone, P = 0.008). Patients with germline BRCA mutations had a median progression-free survival of 19.4 vs. 16.5 months, respectively (P = 0.16) (44). Updated results also suggested an overall survival benefit with cediranib (median 37.8 vs. 23.0 months, P = 0.047) for the BRCA wild-type cohort (71). Hence, the study initially reinforced the hypothesis that the addition of cediranib induced contextual HRD in HR-proficient tumors. In a similar scenario (platinum-sensitive recurrent ovarian cancer), another phase II trial (AVANOVA2) suggested the benefit of adding the VEGF inhibitor bevacizumab to niraparib irrespective of HRD status (median progression-free survival of 11.9 months vs. 5.5 months with niraparib alone for the intention-to-treat population; P < 0.001). Interestingly, once again, the greatest magnitude of benefit with the combination was seen in BRCA wild-type (HR 0.33) and HR-proficient tumors (HR 0.36) compared to BRCA-mutated (HR 0.53) and HRD tumors (HR 0.47) (46).

Subsequently, the phase III GY004 trial compared the combination of cediranib and olaparib versus standard of care platinum-based chemotherapy for platinum-sensitive recurrent ovarian cancer, with negative results (median progression-free survival 10.3 vs. 10.4 months, HR 0.85, 95% CI 0.66 – 1.11). The study also contained an olaparib alone arm, which had a median progression-free survival of 8.2 months. Nevertheless, cediranib plus olaparib and olaparib alone arms were not statistically compared due to hierarchical testing established by the study (50).

Bevacizumab is considered a standard of care option for ovarian cancer in multiple scenarios, including first-line therapy, platinum-sensitive recurrence, and platinum-resistant recurrence. Understanding the role of combining bevacizumab and PARP inhibitors is therefore essential. In the phase III PAOLA trial, the addition of olaparib to bevacizumab was compared with bevacizumab alone as maintenance after first-line therapy. The study was positive, with a significant benefit of the addition of olaparib for the BRCA-mutated and HRD cohorts. Nevertheless, the role of PARP inhibitors is well-established for these subgroups, and the study lacked an olaparib alone arm to clarify if the combination improves outcomes compared to olaparib alone. Moreover, results were disappointing for HR-proficient patients (median progression-free survival 16.6 vs. 16.2 months, HR 1.00, 95% CI 0.75–1.35), the subgroup for which the expectancies for the combination were higher (48).

Currently, several trials are ongoing evaluating PARP inhibitors plus antiangiogenics in different scenarios (**Table 2**). The phase III ICON9 trial is evaluating maintenance with olaparib plus cediranib versus olaparib alone after first-line therapy and may clarify the role of the combination (NCT03278717).

### PARP Inhibitors Plus Chemotherapy

Cytotoxic chemotherapy acts in diverse ways, and their synergy with PARP inhibitors may depend on the mechanism of action of the particular chemotherapeutic agent. Drugs that result in DNA damage, such as platinum and alkylating agents, are interesting options when DNA repair is compromised (72). HRD cells are known to have a high sensitivity to platinum agents. On the other hand, the synergist effect between DNA-damaging agents and PARP inhibitors still needs to be further investigated in clinical trials.

Another possible combination with chemotherapeutic agents is with topoisomerase inhibitors, such as temozolomide and anthracyclines. The topoisomerases I and II participate in DNA replication and repair, controlling DNA topological state. Inhibition of topoisomerase results in replicative stress, jeopardizing DNA repair, which may enhance PARP inhibitor efficacy (73). Through a different mechanism, agents that decrease nucleotides also cause replicative stress. This is the case of the pyrimidine nucleoside analog gemcitabine that inhibits the ribonucleotide reductase, representing another possibility of a combination strategy (74).

Unfortunately, a challenge when using the combination of chemotherapy plus PARP inhibitors is the overlapping toxicities, especially myelosuppression. This limits these combinations or leads to the necessity of substantial dose reductions. In a randomized phase II trial with 162 patients, olaparib combined with carboplatin and paclitaxel, followed by olaparib maintenance, was compared with chemotherapy alone for recurrent platinum-sensitive ovarian cancer. During the combination phase, the olaparib capsule dose was reduced to 200 mg twice daily, instead of 400 mg twice daily as commonly used as maintenance. Although the study showed an improvement in progression-free survival with olaparib, the lack of an olaparib maintenance-only arm precludes conclusions on the role of the combination phase. Additionally, 43% of the patients had grade 3-4 neutropenia in the olaparib plus chemotherapy group (52).

The PARP inhibitor veliparib has a lower PARP trapping activity. Although its potency may be decreased, the drug may be better tolerated, facilitating combination therapies. In the phase III VELIA trial, veliparib was added to carboplatin and paclitaxel as first-line therapy for high-grade serous ovarian carcinoma in a three-arm study. The veliparib-throughout arm received chemotherapy plus veliparib followed by veliparib maintenance, the veliparib combination-only arm received chemotherapy plus veliparib followed by placebo maintenance, and the control arm received chemotherapy plus placebo (concurrent and maintenance). Results showed a benefit in progression-free survival for the veliparib-throughout arm (median 34.7 months vs. 22 months in the control arm; HR 0.44, 95% CI 0.28 – 0.68). On the other hand, the veliparib combination-only arm did not differ from the control arm, and further clarification of the combination role is needed (8).

Regarding other combinations, a phase I/II of veliparib plus topotecan for platinum-resistant or partially platinum-sensitive non-BRCA mutated recurrent ovarian cancer showed disappointing results. No patient had a radiological response, and median progression-free survival was 2.8 months in this poor prognosis population (54). Results were also disappointing in a phase II trial of veliparib plus the alkylating agent cyclophosphamide for previously treated BRCA-mutated ovarian cancer, with an overall response rate of 12% with the combination and 19% with cyclophosphamide alone (53). Another phase I/II study of the veliparib plus topotecan for ovarian cancer is ongoing (NCT01012817). Phase II studies of veliparib plus the alkylating agent temozolomide (NCT01113957) and olaparib plus pegylated liposomal doxorubicin (NCT03161132) for platinum-resistant ovarian cancer are also underway.

### PARP Inhibitors Plus Immune Checkpoint Inhibitors

Initially, the accumulation of neoantigens due to decreased DNA repair in HRD tumors was postulated as the rationale for using immunotherapy in ovarian cancer. Tumors enriched in neoantigens or with a high tumor mutational burden are sensitive to immune checkpoint inhibitors (ICIs). These characteristics are indeed observed when DNA repair is compromised due to deficient mismatch repair, a DNA repair mechanism that corrects mismatched base pairs, insertions, and deletions. However, different from what is observed with deficient mismatch repair/microsatellite instability, alterations in HR do not result in a considerable neoantigen load or high tumor burden (63). Genomic analysis shows that ovarian cancer usually exhibits less than 10 mutations per megabase (75). As previously mentioned, other genomic alterations occur when HRD is present, such as loss of heterozygosity, large-scale state transitions, and telomeric allele imbalance. Facing this, phase II-III studies that evaluated single-agent ICIs have not shown a good efficacy of these drugs in ovarian cancer so far (76–79). Nevertheless, combination therapies may contribute to improving ICIs activity.

Preclinical studies have shown that DNA damage and PARP inhibition stimulate PD-L1 expression (80, 81). High PD-L1 expression is a biomarker that predicts ICIs efficacy in some tumors (82). Another interesting rationale for combining PARP inhibitors and ICIs involves the STING pathway activation. The infiltration of the tumor microenvironment by CD8+ T-cell infiltration seems to be important to the antitumor efficacy of PARP inhibitors. A model of BRCA-mutated triple-negative breast cancer showed that the T-cell recruitment is mediated by cGAS/STING pathway, which is more activated in HRD cells and is enhanced by the addition of PD-L1 blockade. In this model, the function of the STING pathway was required for the efficacy of olaparib alone or combined with ICI (83). Therefore, these data suggest that some synergy might occur with the combination of PARP inhibitors and ICIs.

Phase II trials have already evaluated these combinations for recurrent ovarian cancer. Among platinum-resistant ovarian cancer, overall response rates ranged from 11% to 18% with the combination of olaparib plus durvalumab, olaparib plus dostarlimab plus bevacizumab, and niraparib plus pembrolizumab (55, 58, 59). In a smaller cohort of platinum-resistant recurrent ovarian cancer patients with HRD (n=14), the overall response rate was 35.7% with olaparib plus durvalumab in the multi-arm phase II AMBITION trial (51).

More exciting results were reported for platinum-sensitive ovarian cancer in the MEDIOLA phase II trial. The overall response rate was 71.9% with olaparib plus durvalumab for recurrent platinum-sensitive germline BRCA-mutated ovarian cancer, with a median progression-free survival of 11.1 months (56). For the germline BRCA wild-type population with platinum-sensitive ovarian cancer, olaparib and durvalumab were evaluated with or without bevacizumab. Overall response rates were 34.4% with the doublet regimen and 87.1% with the triplet regimen. Median progression-free survival was 5.5 and 14.7 months, respectively (57).

Nevertheless, these efficacy outcomes still need confirmation in phase III randomized trials, requiring comparison with a PARP inhibitor alone control arm. In that regard, several phase II and III trials evaluating combinations of PARP inhibitor plus ICI are ongoing, especially in the scenarios of maintenance after first-line therapy and platinum-sensitive recurrence (**Table 2**). Results of such trials are eagerly awaited for a better comprehension of the activity of these combinations for ovarian cancer.

## Conclusions

Although PARP inhibitors changed importantly ovarian cancer treatment landscape, some challenges are still faced, especially in the way of improving PARP inhibitor efficacy in HR-proficient tumors and overcoming PARP resistance in HRD tumors. The comprehension of PARP inhibitor resistance mechanisms allows a rationale development of PARP combinations targeting homologous recombination itself, other cross-talking pathways, and cell cycle checkpoints. Some favorable results have been observed in early phase trials of PARP inhibitors combined with drugs targeting ATR, WEE1, and VEGF/VEGFR. Combinations with DNA damaging agents, such as cytotoxic chemotherapy and radiation therapy, are also ongoing, with challenges related to cumulative toxicities. Finally, several trials are investigating if the combination of PARP inhibitors and immunotherapy can improve anti-tumor immune response and enhance treatments’ efficacy. In conclusion, considering all the rationale combinations under evaluation, an optimized and broadened use of PARP inhibitors is expected for the following years.

## Author Contributions

All authors participated in the study conception and design. All authors participated in the literature search, data collection and interpretation, and manuscript writing. RB participated in the construction of figures. All authors reviewed the manuscript and approved the final version. All authors are accountable for all aspects of the work.

## Conflict of Interest

RB has received grant, financial support for educational programs and symposia, and personal fee for expert testimony from AstraZeneca, grant from Novartis, financial support for attending symposia from Roche, and personal fee for expert testimony from Ache, outside the submitted work.

MdPED has received personal fee for expert testimony from AstraZeneca and Novartis, outside the submitted work.

## Publisher’s Note

All claims expressed in this article are solely those of the authors and do not necessarily represent those of their affiliated organizations, or those of the publisher, the editors and the reviewers. Any product that may be evaluated in this article, or claim that may be made by its manufacturer, is not guaranteed or endorsed by the publisher.
